# Efficacy of Stem Cell–Derived Exosomes in Promoting Diabetic Foot Ulcer Healing: A Meta‐Analysis of Preclinical Animal Studies

**DOI:** 10.1155/jdr/2578542

**Published:** 2026-03-17

**Authors:** Xiaona Wang, Lei Pan, Haijun Shen

**Affiliations:** ^1^ Department of General Practice, Jiangsu University, University Hospital (Community Health Service Station), Zhenjiang, Jiangsu, China, ujs.edu.cn; ^2^ Department of Surgery, Zhenjiang First People’s Hospital, Jiangsu University, Clinical Medicine School, Zhenjiang, Jiangsu, China, ujs.edu.cn; ^3^ Department of Preventive Medicine & Public Health Laboratory Science, Jiangsu University, School of Medicine, Zhenjiang, Jiangsu, China, ujs.edu.cn

**Keywords:** diabetic foot ulcer healing, meta-analysis, stem cell–derived exosomes

## Abstract

**Objective:**

The objective of the study is to systematically evaluate the efficacy and mechanisms of action of stem cell–derived exosomes from various sources (including adipose tissue [AD‐MSCs] and bone marrow [BM‐MSCs]) in treating diabetic foot ulcers (DFUs) based on preclinical evidence.

**Method:**

A computerized search was performed across multiple databases including PubMed, CBM, Web of Science, Embase, Wanfang, CNKI, the Cochrane Library, and VIP. The search period covered the period from their inception through December 2024. The search is aimed at identifying all relevant randomized controlled trials (RCTs) using animal models of DFU. Ten studies involving 239 animals were included in the final meta‐analysis. Data synthesis and statistical analysis were performed using RevMan 5.4. The risk of bias was assessed with SYRCLE’s tool.

**Results:**

The pooled results demonstrated that stem cell–derived exosomes significantly promoted wound healing, evidenced by increased wound healing rates on Day 7 (SMD = 8.41, 95% CI: 4.06–12.75, *p* < 0.001; *I* ^2^ = 91*%*) and Day 14 (SMD = 4.89, 95% CI: 3.63–6.14, *p* < 0.001; *I* ^2^ = 0*%*). Furthermore, exosome treatment enhanced neovascularization density (SMD = 7.78, 95% CI: 6.04–9.52, *p* < 0.001), collagen deposition (SMD = 8.59, 95% CI: 2.16–15.03, *p* < 0.05), and reduced levels of inflammatory markers (TNF‐*α* and CD86). Subgroup analyses revealed that the administration dose was a major source of heterogeneity, with a nonlinear dose–response relationship observed. Funnel plots indicated potential publication bias.

**Conclusion:**

Stem cell–derived exosomes represent a highly effective preclinical therapeutic strategy for DFU by accelerating wound closure and improving key wound healing parameters. However, the clinical translation of these findings requires further validation due to the high heterogeneity, potential publication bias, and the inherent limitations of animal studies.

**Trial Registration:**

ClinicalTrials.gov identifier: CRD420251110272

## 1. Research Background

A progressive annual increase has been observed in diabetes incidence globally. In 2017, the global count of individuals with diabetes stood at 451 million, and estimates suggest that this figure may climb to approximately 693 million by the Year 2045 [1]. Diabetes‐related cutaneous ulceration represents a serious long‐term complication, and diabetic foot is the most common presentation. These ulcers heal poorly and are susceptible to infection, which can progress to gangrene. Consequently, they are the principal indication for nontraumatic amputation in routine practice and a major contributor to mortality and disability in diabetes [2]. Epidemiological data indicate that nearly 25% of individuals with diabetes face the potential development of foot ulcers, with such lesions being responsible for roughly 80% of lower extremity amputations associated with the disease [3]. Moreover, the chronic nature of these nonhealing wounds not only leads to considerable deterioration in life quality for affected patients but also places a significant economic strain on healthcare infrastructures. Currently employed clinical strategies, encompassing hyperbaric oxygen therapy, negative pressure wound therapy, and surgical revascularization, have demonstrated only restricted effectiveness in managing this condition [4]. To this day, diabetic skin ulcers remain a challenging medical problem, making the exploration of effective treatment methods an urgent priority.

Stem cells provide distinct benefits for wound repair because self‐renewal sustains granulation tissue formation and vascular regeneration; the contribution of stem cells to healing has received growing attention [5]. Exosomes (Exos) are paracrine cellular products, namely, nanoscale vesicles (30–150 nm in diameter) enclosed by a double‐layer lipid membrane. Exos are enriched in proteins, nucleic acids, and endogenous factors; they convey the biological information of parent cells and recapitulate many parent cell functions [6]. Stem cell–derived Exos are microvesicles released via paracrine signaling by stem cells, and they display stem cell surface markers and chemokines. These vesicles encapsulate bioactive substances generated during stem cell growth and proliferation, including nucleic acid fragments, peptide chains, cytokines, and enzymes [7]. As products that reflect stem cell activity, stem cell–derived Exos exhibit biological functions similar to those of stem cells and present notable clinical advantages [8], including relatively low immunogenicity and a reduced risk of tumorigenesis [9]. Of note, Exos derived from mesenchymal stem cells (MSCs) of different tissue origins—such as adipose tissue, bone marrow, and umbilical cord—may differ in terms of yield, molecular cargo, and biological functions. One study [10] indicated that adipose tissue–derived MSCs (AD‐MSCs), being a more prolific and accessible source than bone marrow–derived MSCs (BM‐MSCs), are likely to secrete a greater quantity of extracellular vesicles, with their Exos demonstrating a robust ability to promote angiogenesis. In contrast, BM‐MSC‐derived Exos have demonstrated advantages in modulating specific immune cell responses, such as suppressing profibrotic Th17 cells and promoting the expansion of regulatory T cells (Tregs) [11]. These source‐dependent variations may ultimately influence their therapeutic outcomes in diabetic wound healing. The therapeutic benefits of Exos are largely attributed to their rich cargo, particularly microRNAs (miRNAs). These miRNAs can be delivered to target cells in the wound microenvironment—such as keratinocytes, fibroblasts, and endothelial cells—where they coordinate multiple stages of wound repair by regulating gene expression. Furthermore, miR‐125b‐5p has been shown to enhance the repair of diabetic hind limb ischemia by targeting and inhibiting ACER2, thereby promoting the proliferation and migration of ischemic muscle cells [12]. Exos can also carry specific molecules such as miR‐146a to inhibit TRAF6/IRAK1 and block the NF‐*κ*B pathway, subsequently reducing the release of proinflammatory factors like TNF‐*α*, IL‐6, and IL‐1*β*, ultimately achieving an anti‐inflammatory effect by suppressing excessive immune responses. Additionally, other components in Exos, such as the TSG‐6 protein, can further reshape the immune microenvironment by inducing M2 macrophage polarization and the formation of Tregs, which helps alleviate the chronic inflammation typically observed in diabetic wounds [13]. Accordingly, stem cell–derived Exos have emerged as a promising focus in regenerative medicine.

The primary objective of this research is to evaluate the therapeutic potential of Exos derived from stem cells in enhancing the wound repair process associated with diabetic ulceration. Given the scarcity of clinical case reports, preclinical animal studies utilizing Exos in models of diabetic ulceration were included in the meta‐analysis. The goal is to establish a theoretical foundation for the clinical management of diabetic ulcers.

## 2. Research Methods

This study has been registered in the International Prospective Register of Systematic Reviews (PROSPERO).

### 2.1. Information Retrieval

Two researchers searched Chinese databases (CNKI, Wanfang, VIP, and CBM) and English databases (PubMed, Web of Science, Embase, and Cochrane Library). Keywords included “stem cell‐derived exosomes,” “diabetic foot ulcer,” “wound healing,” etc. A comprehensive search strategy, incorporating both controlled vocabulary and free‐text terms, was implemented across the electronic databases. The retrieval timeframe extended from the earliest records available in each database through December 2024. Manual searching of the reference lists from all included articles and relevant review articles was performed. Key search concepts comprised terminology related to “exosomes” combined with descriptors for “stem cells” (including “Cells, Stem” and “stem cell”) and terms denoting “diabetic foot” (such as “Foot Ulcer” and “diabetic foot ulcer,” among others).

### 2.2. Inclusion and Exclusion Criteria

The inclusion criteria encompassed all randomized controlled animal studies investigating the application of stem cell–derived Exos for enhancing the healing process of diabetic ulcer wounds, without imposing any language restrictions.

The inclusion criteria are as follows: (1) study type: controlled animal studies (with random allocation), with no restrictions on language or publication type; (2) animal experiments, with no restrictions on species, gender, or age; and (3) interventions involving the use of Exo‐based therapy in the full‐thickness excisional diabetic wound model.

The exclusion criteria are as follows: (1) nonrandomized controlled trials and quasirandomized controlled trials (e.g., those using allocation by date of birth and alternate cases), (2) nondiabetic models, (3) nonskin wounds, (4) non–stem cell–derived Exos, (5) interventions do not meet the criteria, (6) duplicate publications, (7) insufficient original data for evaluation, and (8) insufficient extractable data for statistical analysis.

### 2.3. Study Selection, Data Extraction, and Outcome Measures

Two researchers (Xiaona Wang and Lei Pan) independently performed the tasks of literature retrieval, study selection, and data extraction in accordance with pre‐established eligibility criteria. They screened the literature and extracted information using a predesigned form. Any disagreements were resolved by a third‐party adjudicator. Data collection encompassed the following investigational parameters: titles of the selected articles, year of publication, lead author names, number of participants, interventional approaches, comparator group protocols, key efficacy endpoints, and additional pertinent information. The extracted data were systematically organized in an Excel spreadsheet. For data presented exclusively in graphical form, numerical values were accurately extracted using GetData Graph Digitizer software, Version 2.26.

The experimental group was treated with Exos derived from stem cells in animal models of diabetes. No limitations were imposed on factors such as animal species, intervention techniques, treatment duration, dosage, carrier type, wound dimensions, injection intervals, or sites of injection. In contrast, the control group was administered nonfunctional solutions like PBS buffer or saline.

The outcome measures are as follows:

1. Wound healing rate
2. Neovascularization density
3. Collagen fiber deposition percentage
4. Inflammatory markers

### 2.4. Quality Evaluation

The methodological quality of the included animal studies was assessed using SYRCLE’s risk of bias tool [14], which is specifically designed for systematic reviews of animal studies. The completed assessment, which details the domain‐specific judgments (for sequence generation, blinding, etc.) for every study.

#### 2.4.1. Statistical Analysis

Statistical synthesis was performed using RevMan (Version 5.4). For continuous outcomes, the pooled effect was calculated as the standardized mean difference (SMD) with 95% confidence interval (CI). For dichotomous outcomes, the risk ratio (RR) or mean difference (MD) with 95% CI was used. Between‐study heterogeneity was quantified using the *I* ^2^ statistic. A fixed‐effects model was applied when heterogeneity was low (*I* ^2^ ≤ 50*%*); otherwise, a random‐effects model was used (*I* ^2^ > 50*%*). Statistical significance was set at *p* < 0.05. Potential publication bias was assessed by funnel plot inspection, and sensitivity analyses were conducted to evaluate the robustness of the results.

## 3. Results

### 3.1. Literature Screening

**Figure 1:**
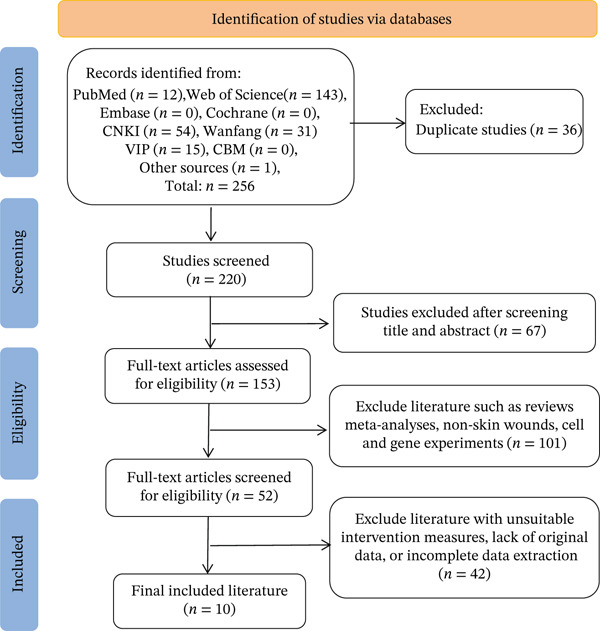
Literature search process.

**Figure 2:**
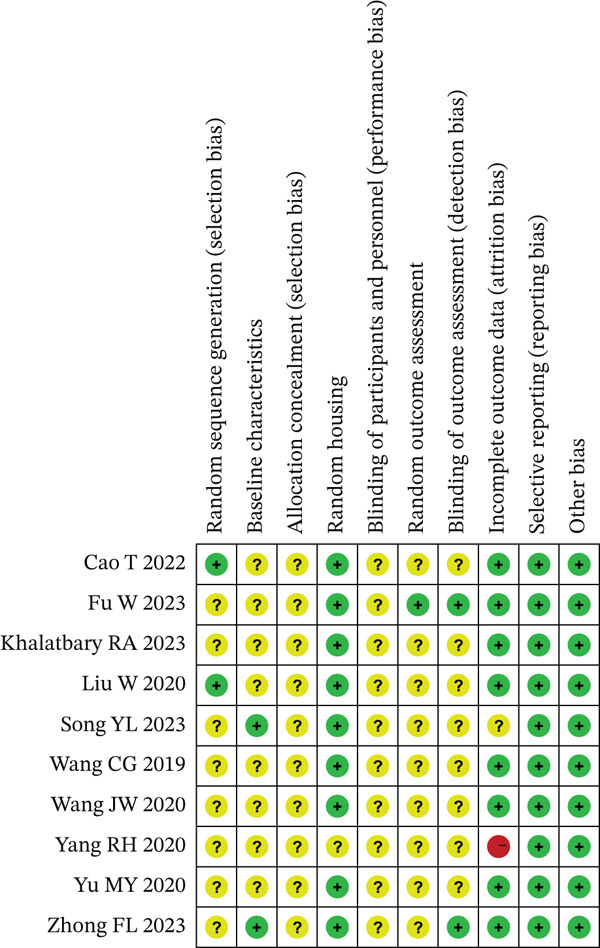
Summary of risk of bias assessment for included studies.

Domestic and international databases were systematically queried using standardized Chinese‐ and English‐language terms. The search identified 256 records (Chinese *n* = 100; English *n* = 156). After deduplication, 220 unique records remained. Screening of titles and abstracts yielded 52 candidates, and full‐text assessment ultimately retained 10 studies [15–24], including 5 Chinese and 5 English publications. The search flow is shown in Figure 1. The basic characteristics of the included studies are summarized in Table 1, and the risk of bias assessment is presented in Figure 2.

**Table 1:** Inclusion of basic literature characteristics.

First author	Publication date	Species	Sources of exosomes	Purification method	Wound form	Diameter or area	Experimental group	Control group	Method	
									Experimental group	Control group
Zhong [15]	2023	C57BL/6 mice	AD‐MSCs	Ultracentrifugation	Cut	10 mm	12	12	Subcutaneous injection; 70 *μ*g/100 *μ*L	PBS
Cao [16]	2022	Kunming mice	AD‐MSCs	Density gradient centrifugation	Cut	10 mm	6	6	Intradermal injection; 100 *μ*g/100 *μ*L	PBS
Song [17]	2023	BALB/c mice	AD‐MSCs	Ultracentrifugation	Cut	10 mm	6	6	Local injection; 100 *μ*g/100 *μ*L	PBS
Wang [18]	2020	BALB/c mice	AD‐MSCs	Density gradient centrifugation	Cut	8 mm	12	12	Intradermal injection; 100 *μ*g/100 *μ*L	PBS
Fu [19]	2023	Rats	AD‐MSCs	Ultracentrifugation	Cut	15 mm	15	15	Tail vein injection; 200 *μ*g/200 *μ*L	PBS
Khalatbary [20]	2023	Wistar rats	AD‐MSCs	Ultracentrifugation	Cut	15 mm	15	15	Subcutaneous injection; 200 *μ*g/200 *μ*L	PBS
Liu [21]	2020	SD rats	BM‐MSCs	Ultracentrifugation	Cut	20 mm	18	18	Subcutaneous injection; 100 *μ*g/100 *μ*L	PBS
Yu [22]	2020	SD rats	BM‐MSCs	Ultracentrifugation	Cut	20 mm	10	10	Multipoint injection; NR	PBS
Wang [23]	2019	ICR mice	AD‐MSCs	Ultracentrifugation	Cut	8 mm∗2	12	12	Subcutaneous injection; 10 *μ*g	NS
Yang [24]	2020	SD rats	BM‐MSCs	Ultracentrifugation	Scald	Rat dorsum 30% surface area	14	13	Tail vein injection; 200 *μ*g/mL	PBS

Abbreviation: NR, not reported in the original study.

### 3.2. Result Analysis

#### 3.2.1. Wound Healing Rate

**Figure 3:**
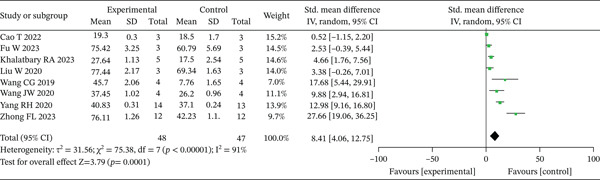
Meta‐analysis of Day 7 wound healing rates in the experimental versus control groups.

Eight eligible trials [15, 16, 18–21, 23, 24] documented the wound healing rate measured on Day 7. Significant heterogeneity was detected across these studies (*p* < 0.10, *I* ^2^ > 50*%*), prompting the application of a random‐effects model for analysis. Pooled results demonstrated significantly accelerated wound healing in the experimental group compared to controls at the 7‐day assessment point (SMD = 8.41, 95% CI: 4.06–12.75, *p* < 0.001), as visually presented in Figure 3.

**Figure 4:**
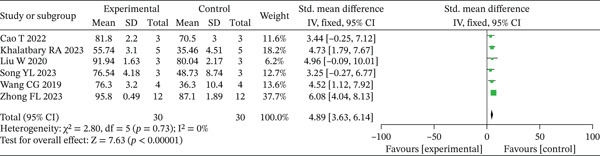
Meta‐analysis of Day 14 wound healing in the experimental versus control groups.

Day 14 wound healing rates were documented in six investigations [15–17, 20, 21, 23]. As minimal heterogeneity was observed among these trials (*p* > 0.10, *I* ^2^ < 50*%*), a fixed‐effects model was employed for statistical synthesis. The combined results revealed significantly enhanced wound healing progression in the experimental group compared to controls at the 14‐day evaluation point (SMD = 4.89, 95% CI: 3.63–6.14, *p* < 0.001), with these outcomes graphically represented in Figure 4. Sensitivity analyses, which excluded one study at a time, did not change the statistical significance or the direction of the present findings.

#### 3.2.2. Neovascularization Density

**Figure 5:**
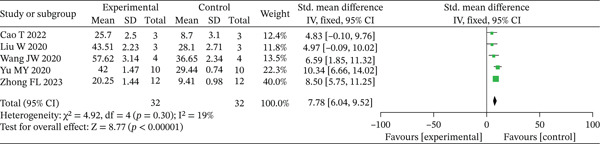
Meta‐analysis of the neovascularization density on Day 14 between the experimental and control groups.

Five research reports [15, 16, 18, 21, 22] documented the status of wound neovascularization evaluated at Day 14 postintervention. The assessment of neovascularization was consistently performed in all five studies through immunohistochemical staining employing the CD31 endothelial cell marker. Due to the consistent findings across these studies (*p* > 0.10, *I* ^2^ < 50*%*), a fixed‐effects analytical model was implemented. The pooled analysis demonstrated significantly greater neovascularization density in the experimental group relative to controls at the 14‐day assessment point (SMD = 7.78, 95% CI: 6.04–9.52, *p* < 0.001), with visual representation provided in Figure 5.

Sensitivity analyses, which excluded one study at a time, did not change the statistical significance or the direction of the present findings.

#### 3.2.3. Collagen Fiber Deposition

**Figure 6:**
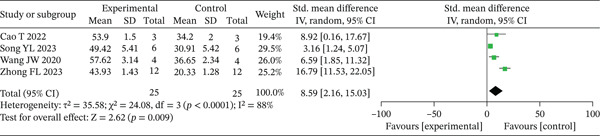
Meta‐analysis of collagen volume fraction on Day 14 in the experimental group and control group.

Collagen fiber deposition was reported in four studies [15–18] on Day 14, with observed heterogeneity (*p* < 0.10, *I* ^2^ > 50*%*). A random‐effects model was applied for the analysis. The meta‐analysis revealed a higher collagen volume fraction percentage in the experimental group compared to the control group at Day 14 (SMD = 8.59, 95% CI: 2.16–15.03, *p* < 0.05), as illustrated in Figure 6. However, it is important to note that this result exhibited high heterogeneity (*I* ^2^ = 88*%*), indicating considerable variation in effect sizes across studies. Therefore, this pooled result should be considered exploratory.

We performed a sensitivity analysis using the leave‐one‐out method to assess the robustness of the pooled results. The analysis indicated that the robustness of the findings was somewhat influenced by individual studies with high heterogeneity. Specifically, after excluding the study by Wang et al. [18], the heterogeneity among studies significantly decreased (*I* ^2^ from 88% to 35%), while the pooled effect size remained highly statistically significant (*p* = 0.002). This finding suggests that, after removing specific sources of heterogeneity, the remaining studies support a more consistent and robust positive effect. However, it should also be noted that when the study by Zhong et al. [15] was excluded, the effect size became only marginally significant (*p* = 0.06), reflecting that the current evidence base is still insufficient. Therefore, more homogeneous, high‐quality studies are needed in the future to further validate this conclusion.

#### 3.2.4. Inflammatory Markers

**Figure 7:**
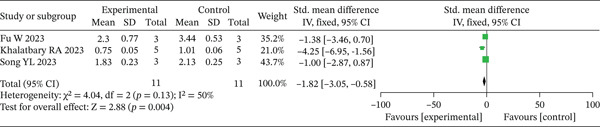
Meta‐analysis of TNF‐*α* expression in the experimental group versus the control group.

**Figure 8:**
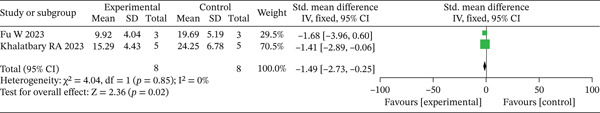
Meta‐analysis of CD86 expression in the experimental group versus the control group.

Inflammatory markers were qualitatively synthesized due to the limited number of available studies. Data from three independent investigations [17, 19, 20] consistently reported a reduction in TNF‐*α* expression in the experimental group compared to controls. Similarly, both studies examining CD86 expression [19, 20] reported consistently lower levels under experimental conditions. The direction of effect for both markers was uniform across all included studies, suggesting a potential trend toward the suppression of proinflammatory responses, though this conclusion is constrained by the small sample of literature. The forest plots for TNF‐*α* and CD86 (Figures 7 and 8, respectively) are presented for illustrative purposes.

### 3.3. Publication Bias

**Figure 9:**
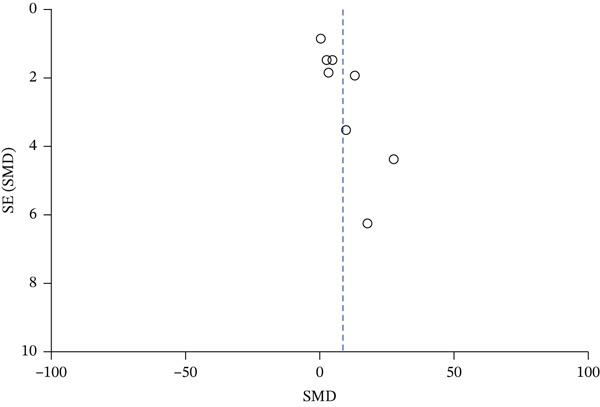
Publication bias.

The results showed an asymmetrical distribution of data in the funnel plot (Figure 9). In addition to the visual inspection of the funnel plot, publication bias was quantitatively assessed. Begg’s rank correlation test indicated potential bias (*Z* = 2.47, *p* = 0.013). Similarly, Egger’s linear regression test showed a significant intercept (bias coefficient = 5.86, *t* = 4.52, *p* = 0.004), providing strong statistical evidence for the presence of publication bias. This asymmetry may be attributed to the fact that most published literature tends to favor reporting positive outcomes, suggesting a potential risk of publication bias among the included studies.

### 3.4. Subgroup Analysis

#### 3.4.1. Overall Effect Analysis

A total of eight studies measuring the wound healing rate on Day 7 were included in the meta‐analysis. The overall pooled analysis showed that compared to the control group, stem cell–derived Exos demonstrated a significant positive effect on improving the wound healing rate (test for overall effect: *Z* = 3.79, *p* = 0.0001). However, substantial heterogeneity was observed among the studies (*I* ^2^ = 91*%*, *p* < 0.00001), prompting subgroup analyses to explore potential sources of this heterogeneity.

#### 3.4.2. Subgroup Analysis

##### 3.4.2.1. Subgroup Analysis Based on Exo Source.

Studies were divided into two subgroups based on the Exo source: adipose‐derived MSC exosomes (ADMSC‐ex) and BM‐MSC Exos. The analysis revealed that the AD‐MSC subgroup had a statistically significant therapeutic effect (*Z* = 3.29, *p* = 0.001), whereas the BM‐MSC subgroup showed a positive trend that did not reach statistical significance (*Z* = 1.70, *p* = 0.09). High heterogeneity was present within both subgroups (*I* ^2^ > 90*%*). The test for subgroup differences was not statistically significant (chi^2^ = 0.00, *p* = 0.96, *I* ^2^ = 0*%*), indicating that the cellular origin of the Exos might not be a major source of the overall heterogeneity.

##### 3.4.2.2. Subgroup Analysis Based on Animal Species.

To investigate the influence of the animal model, studies were categorized into rat and mouse subgroups. The results indicated that stem cell Exos exhibited a significant therapeutic effect in both rat models (*Z* = 2.66, *p* = 0.008) and mouse models (*Z* = 2.04, *p* = 0.04). Heterogeneity remained high within both subgroups (*I* ^2^ = 85*%* and 94%, respectively). The test for subgroup differences was not significant (*p* = 0.27), suggesting consistency of the therapeutic effect across different species among the studies included in this analysis.

##### 3.4.2.3. Subgroup Analysis Based on Administration Dose.

Subgroup analysis was performed based on the relative dose of Exos, categorized into low‐, medium‐, and high‐dose subgroups. The results suggested a potential nonlinear relationship between treatment effect and dose: Both the low‐dose group (*Z* = 4.84, *p* < 0.00001) and the high‐dose group (*Z* = 2.28, *p* = 0.02) showed significant therapeutic effects, whereas the effect in the medium‐dose group was not statistically significant (*Z* = 1.61, *p* = 0.11). Notably, the differences between subgroups were highly statistically significant (chi^2^ = 14.29, *p* = 0.0008, *I* ^2^ = 86.0*%*), strongly suggesting that the administration dose is an important source of heterogeneity in this study. Furthermore, the low‐dose subgroup exhibited the lowest heterogeneity (*I* ^2^ = 42*%*), indicating the most stable results at this dose level.

## 4. Discussion

Exos originating from stem cells serve as crucial mediators in cellular communication, transporting genetic material from their parent cells and delivering specific molecular constituents to recipient cells, thereby retaining numerous therapeutic advantages characteristic of stem cell–based treatments. These biologically active vesicles potentially demonstrate enhanced efficacy and greater therapeutic versatility compared to Exos derived from conventional cell types. Furthermore, they present distinct benefits regarding application breadth, precise dosage administration, and reduced immunogenic reactions when contrasted with unmodified stem cell therapies [25]. Research indicates that Exos obtained from adipose‐derived stem cells (ADSCs‐Exos) could facilitate cutaneous wound repair through mechanisms including inflammation regulation, stimulation of cellular proliferation, migration, differentiation, vascular formation, and extracellular matrix reorganization, thereby offering novel insights and methodological approaches for managing skin wound healing [26].

Findings from the meta‐analysis demonstrate that relative to control conditions, administration of Exos originating from stem cells to the treatment group resulted in significantly elevated wound healing rate on both Day 7 and Day 14 posttreatment in diabetic ulcer models. Moreover, the treatment led to an increase in neovascularization at the wound site, enhanced collagen fiber deposition, decreased levels of inflammatory markers, and further accelerated wound healing. These results suggest that Exos derived from stem cells promote wound repair through mechanisms such as angiogenesis, collagen deposition, and inflammation reduction. This process enhances local blood circulation and oxygenation, stimulates granulation tissue formation, and accelerates the healing of diabetic ulcers.

Recent years have witnessed growing research interest in Exos derived from stem cells for managing DFU. Investigations demonstrate that exosomal vesicles obtained from bone marrow and umbilical cord MSCs enhance DFU outcomes through facilitating wound repair processes and attenuating inflammatory reactions [8, 27]. MSC‐Exos contribute to diabetic ulcer recovery by regulating the wound’s inflammatory microenvironment, stimulating vascular formation, and suppressing apoptosis induced by oxidative stress [28]. According to research findings [29–31], ADMSC‐ex can inhibit high glucose‐stimulated neutrophil extracellular trap (NET) generation and modulate both NET formation and related inflammatory cascades via the TRPV4 signaling pathway, thereby establishing novel theoretical foundations for ADMSC‐ex utilization in DFU therapy.

This discovery partially elucidates that wound healing depends not merely on regulating proliferative and remodeling phases of cutaneous recovery but also on inflammatory phase modulation [32], which corresponds with the conclusions drawn in the current investigation.

This meta‐analysis confirms the significant therapeutic effect of stem cell–derived Exos on diabetic wound healing in animal models, though substantial heterogeneity (*I* ^2^ = 91*%*) was observed. It is noteworthy that the analysis revealed unusually high effect sizes (e.g., SMD = 8.41). These substantial values are likely attributable to the influence of small study effects, as the limited sample sizes typical of preclinical research are known to inflate effect estimates. Furthermore, the potential for publication bias in this emerging field, where studies with positive results are more likely to be published, may also contribute to the observed magnitude. Subgroup analyses provided important insights into potential sources of this variability.

Analysis by Exo source showed that while ADSC‐derived Exos demonstrated statistically significant effects, BMSC‐derived Exos also exhibited a positive trend. The absence of significant subgroup differences suggests that Exo source may not be a major factor influencing therapeutic efficacy, indicating possible conserved healing mechanisms across MSC types. A notable finding was the nonlinear dose–response relationship. Both low and high Exo doses significantly improved wound healing, whereas the medium dose showed no significant effect. This U‐shaped response pattern may indicate activation of different biological pathways at varying concentrations. The low heterogeneity in the low‐dose subgroup (*I* ^2^ = 42*%*) further suggests that this may represent a more consistent therapeutic window. The consistent therapeutic effects observed across both rat and mouse models strengthen the generalizability of our findings and support the biological plausibility of Exo‐mediated wound healing across species.

The findings of this meta‐analysis must be interpreted in the context of its limitations. Firstly, the total number of animals across all included studies was relatively small (*n* = 239), which limits the statistical power and precision of our effect estimates and cautions against overgeneralization of the results. Secondly, we observed significant heterogeneity in Exo preparation protocols (e.g., isolation methods and dosing regimens), posing challenges for direct comparison and highlighting a key translational obstacle. Thirdly, all evidence comes from preclinical animal models that cannot fully replicate human disease complexity, creating a notable translational gap. Beyond these specific limitations, the translational path for Exo therapy faces universal barriers, including the development of scalable, GMP‐compliant production processes and standardized safety, and dosing profiles. These considerations collectively underscore the need for comprehensive evaluation in future trials.

## 5. Conclusion

While our meta‐analysis suggests a potential benefit, the overall certainty of the evidence remains limited as assessed by the GRADE approach. Consequently, we have taken care to avoid overstating the clinical implications in the Conclusion section. Given these limitations, the therapeutic potential of stem cell–derived Exos for diabetic wound healing, while promising, requires further validation through well‐designed clinical studies before any clinical applications can be considered, underscoring the need for robust future research.

## Author Contributions

Xiaona Wang: conceptualization, methodology, literature search, formal analysis, data curation, and writing—original draft preparation. Lei Pan: literature search, study screening, risk of bias assessment, data extraction, validation, and writing—review and editing. Haijun Shen: supervision, project administration, resources, funding acquisition, methodology, and writing—review and editing.

## Funding

The study was funded by the National Natural Science Foundation of China (10.13039/501100001809, 81402870).

## Disclosure

All authors have read and agreed to the final version of the manuscript.

## Conflicts of Interest

The authors declare no conflicts of interest.

## Data Availability

The data that support the findings of this study are available from the corresponding author upon reasonable request.
